# Differential Expression of Innate and Adaptive Immune Genes during Acute Physical Exercise in American Quarter Horses

**DOI:** 10.3390/ani13020308

**Published:** 2023-01-16

**Authors:** Judith Wilson, Marcos De Donato, Brooke Appelbaum, Carly Turner Garcia, Sunday Peters

**Affiliations:** 1 Department of Animal Science, Berry College, Mount Berry, GA 30149, USA; 2 Tecnologico de Monterrey, Escuela de Ingenierías y Ciencias, Querétaro, CP 76130, Mexico; 3 The Center for Aquaculture Technologies, San Diego, CA 92121, USA; 4 Lazy E Ranch, Guthrie, OK 73044, USA

**Keywords:** differential expression, acute exercise, inflammation, overtraining syndrome

## Abstract

**Simple Summary:**

Overtraining syndrome (OTS) is the reduction in performance due to excess training and lack of proper recovery, which can lead to the chronic deprivation of energy and a reduction in the repair of small damages that accumulate over time. At the early stages, OTS can be presented as functional (FOR), nonfunctional overreaching (NFOR), or full overtraining syndrome, with no clear limits among the three stages. Here, the effect of acute, intense physical exercise on the expression of innate and adaptive immune genes was assessed and a strategy was developed to evaluate any indication of OTS. The three main pathways containing genes that were affected by acute, intense physical exercise were Th1 and Th2 cell differentiation, and the NF-kappa B and chemokine signaling pathways, suggesting the activation of proinflammatory responses as the result of the stress from the acute exercise. Expression analysis of key genes could be used to evaluate the effectiveness of the training scheme in horses so that the best performance can be achieved in high-performance athletes without the risk of developing OTS.

**Abstract:**

Overtraining syndrome (OTS) is the reduction in performance due to excess training and lack of proper recovery, which can lead to a chronic deprivation of energy and reduction in the repair of damage that can accumulate over time. Here, the effect of acute, intense physical exercise on the expression of innate and adaptive immune genes in 12 racing-bred American Quarter Horses, after resting for 3 days and immediately after intense exercise for 1.8 miles were compared. The expression of 84 genes related to innate and adaptive immune responses was analyzed. Significant variation among individuals and between sexes was observed. The analysis showed that five genes were differentially expressed in both females and males, three only in females, and two in males. The upregulated genes were IL13 (male only), CCR4 (female only), TLR6, TLR9 (female only), NFKBIA, CXCR3, and TLR4, while the downregulated genes were IL6 (female only), CD4 (male only), and MYD88. The three main pathways containing genes that were affected by acute, intense physical exercise were Th1 and Th2 cell differentiation, and the NF-kappa B and chemokine signaling pathways, suggesting the activation of the proinflammatory responses as a result of the stress from the acute exercise. Gene expression could be used to assess indications of OTS.

## 1. Introduction

According to the American Horse Council Foundation’s 2017 horse economy study, the horse industry contributes USD 122 billion to the U.S. economy each year and employs 1.74 million people [https://www.horsecouncil.org/resources/economics/, accessed on 5 July 2022]. The American Quarter Horse is the most popular breed in the U.S., having 2.5 of the 7.2 million horses in the country [1].

Overtraining syndrome (OTS) caused by excess training and the lack of proper recovery as well as the stress caused by endurance or competition can lead to temporary immunosuppression in athletes, which has been termed as an ‘open window’ opportunity for infection, placing them at a greater risk [2]. In this sense, Pedersen et al. [3] and Walsh and Oliver [4] carried out literature reviews and reported that due to changes in immune function, numerous studies suggested that moderate exercise may reduce the incidence of upper respiratory tract infections. However, it has also been suggested that intense training or intense competition may increase this kind of infection.

Sakharov et al. [5] studied how training intensity could change the gene expression by subjecting skiers to an exhausting treadmill test (RTE) or a moderate treadmill test (MT). RTE resulted in 310 genes upregulated, while only 69 genes were upregulated after MT, indicating a greater change in gene expression in response to strenuous exercise. The implicated genes were involved in a variety of known pathways related to inflammation, stress response, signal transduction, and apoptosis.

Horses are an excellent model organism for studies into the genetic response to exercise-induced stress because of their intrinsic capacity for athletic performance and the generally uniform nature of their genetic and environmental backgrounds [6]. However, insufficient studies have been carried out on horses to be able to understand the causative effects of exercise-induced stress in some of the gene pathways related to the immune system.

The immune system provides an immediate response of the host to react against both infectious and non-infectious stimuli, making it a good sensor for monitoring the health and welfare of the organism, and allowing for the detection of adaptative failure, abnormal behavior, and poor welfare [7]. A study by Büttner et al. [8] showed that an exhaustive treadmill test produced the upregulation of 450 genes and the downregulation of 150 genes, whose functions were associated with response to stress and inflammatory response. The genes for the stress (heat shock) proteins HSPA1A and HSPH1 and for the matrix metalloproteinase MMP-9 showed the most prominent increases, whereas the YES1 oncogene (YES1) and CD160 (BY55) were most strongly reduced.

Interleukin-6 (IL6) gene expression was examined in humans and horses by Capomaccio et al. [9], who discovered that highly trained humans and horses expressed this gene more significantly than in the untrained participants. The same group [10] examined the long-term metabolic and biomolecular alterations in young Thoroughbred horses during their first four months of training and found changes in the expression of six genes: interleukin-4 (IL4), interleukin-6 (IL6), interleukin-10 (IL10), interleukin-1β (IL1B), octamer-binding transcription factor 1 (POU class 2 homeobox 1, POU2F1), and B-cell lymphoma/leukemia 11A (BCL11A). Among the six genes studied, they found significant upregulation in IL4, IL6, and POU2F1, while BCL11A was downregulated at the end of the training period.

In a subsequent study, Capelli et al. [7] investigated the immunological parameters in horses at rest and during the first three months of incremental sprint exercise training to evaluate its effect on the immunological status. The expression of 10 genes—interleukin 1β (IL1B), interleukin 4 (IL4), interleukin 6 (IL6), interleukin 2 (IL2), interleukin 3 (IL3), interleukin 5 (IL5), interleukin 8 (IL8), transforming growth factor β and α (TGFB), tumor necrosis factor α (TNF), and interferon γ (IFNG)—was analyzed and showed that the IL-2, IL-4, and IL-8 genes were significantly upregulated at both 30 days (T30) and 90 days (T90) after the start of training, with respect to the time prior to training (T0). In addition, TGFB and IL5 were significantly downregulated at T30 and T90, respectively, while IL1B and IL3 expression was strongly augmented at the same time. These results showed the effect of the aggregation of complex stressful stimuli on some genes of the innate immune system.

Capomaccio et al. [6], 10 years ago, carried out an RNA-Seq experiment using SOLiD technology to monitor the transcriptional architecture by comparing the gene expression levels in animals at rest and after competition. They found that the competition-induced stress strongly modulated the genes CCL4, CCL5, CD4, interleukins (IL8, IL18), IL22BP, TLR, CXCR4, integrins (ITGAL and ITGAM), kinases (MAP3K4 and MAPK14), and metalloproteinases (MMP1, MMP8, MMP25, and MMP27). However, the solid technology and the low depth of sequencing did not allow for further elucidation of the details of the splicing variants or the quantification of the expression of lowly expressed genes.

Few studies have looked at the impact of exercise-induced stress on the innate and adaptive immune systems in horses, despite the significance of stress on the immune system and on the health of the organism. This study was therefore conducted to find out how acute, severe exercise affects the expression of innate and adaptive immune genes in American Quarter Horses.

## 2. Materials and Methods

### 2.1. Animal Sampling

Twelve racing-bred American Quarter Horses between 22 and 26 months of age, six colts and six fillies, were used in the current study. All horses were in good health with a body condition score of 5, according to the scoring method by Henneke et al. [11]. The horses were all from same farm. Routine preventive health care was provided to all horses (deworming, vaccinations). The horses were given alfalfa hay and an Omolene blend concentrate twice daily. This study was approved by the Institutional Animal Care and Use Committee (IACUC) of Berry College with approval number 002-2021.

The horses were ridden for 5 consecutive days with 2 consecutive days of rest a week, as a standard training. To obtain the blood samples, horses were rested for 3 consecutive days prior to blood sampling. Samples were obtained from 7:00–7:30 am, 2 h after the feeding, and they were given 30 s after haltering and standing quietly to acclimate to the handler prior to sample collection. Samples were collected into Tempus™ Blood RNA (ThermoFisher Scientific, Waltham, MA, USA) vacutainer tubes. Prior to exercise, horses were briefly groomed, tacked up, and walked either solo or with a pony horse to the track (approximately 350 yards). After that, the horses were galloped for 1.2 miles on a dirt track by two jockeys of similar build, they were then jogged for 0.6 miles to the end of the track and back to the barn. Upon entrance to the barn, the horses were immediately untacked, and blood was drawn post exercise. The horses were then cooled down for 30 min on a lead walker before returning to their stalls. No horses were stressed by venipuncture and each sample was obtained on the first attempt.

### 2.2. RNA Extraction and qPCR

Blood samples in the Tempus tubes were stored at 4 °C until use for RNA extraction within the next 4 days. For the RNA extraction, the Stabilized Blood-to-CT™ Nucleic Acid Preparation Kit for qPCR (ThermoFisher Scientific) was used according to the recommendations of the manufacturer. These samples were stored at −80 °C until RT-qPCR could be carried out.

The RT2 Profiler equine PCR Array (PAEC-052ZA; Qiagen) was used in accordance with the manufacturer’s instructions to analyze the expression of 84 genes linked to innate and adaptive immune responses. Briefly, RT2 SYBR Green ROX qPCR Mastermix was used for cDNA synthesis. Then, a 25 μL reaction volume was run in the 96-well plate with PCR primers of the arrays. The array contained 84 adaptive and innate immune-related genes as well as five housekeeping genes (β-actin, glyceraldehyde-3-phosphate dehydrogenase, hypoxanthine phosphoribosyltransferase 1, TATA box binding protein, and tyrosine 3-monooxygenase), one genomic DNA control to detect gDNA contamination, three reverse transcription controls to quality-check for impurities that may degrade RNA, and three positive PCR controls to verify the efficiency of the PCR amplification of the cDNA template. Real-time PCR was carried out on a QuantStudio 7 Flex Real-Time PCR System (Applied Biosystems) using the following cycling conditions: 95 °C for 10 min, 40 cycles of denaturation at 95 °C for 15 s, and 60 °C for 1 min.

### 2.3. Data Analysis

For the analysis of the gene expression, real-time data were normalized with the average Ct values of the housekeeping genes. The fold change in gene expression was calculated using the $2^{-\Delta \Delta C_{T}}$ method [12], using as a control, the normalized expression of the individuals before the exercise and as treatment, after the exercise. The Student’s *t*-test was used to determine the statistical significance of the fold change in genes expressed by comparing the animals before and after the exercise and also to check if there was a difference between the colts and fillies before and after the exercise.

The volcano plot was constructed in Excel using the log 2 transformation of the fold change calculated using the $2^{-\Delta \Delta C_{T}}$ method and the log 10 of the *p*-value of the Student’s *t*-test. The heatmap was constructed using the RT² Profiler PCR Data Analysis pipeline at the GeneGlobe Data Analysis Center from Qiagen (https://geneglobe.qiagen.com/us/analyze, accessed on 12 October 2022). The protein networks of the differentially expressed genes were obtained from the STRING database (https://string-db.org/), which generates functional enrichment analysis of protein–protein interaction networks from evidence compiled from the co-expression analysis, experimental data, associations from the databases, and the published data.

## 3. Results

### 3.1. Normalization

In this study, we examined the effects of exercise-induced stress on the expression of 84 genes implicated in the innate and adaptive immune response in horses by comparing the status of the genes before and after the exercise, using the real-time PCR array (from Qiagen), which is the gold standard for analyzing gene expression. For the normalization of the expression level in each sample, we used the housekeeping genes: beta actin (ACTB), beta-2-microglobulin (B2M), glyceraldehyde-3-phosphate dehydrogenase (GAPDH), hypoxanthine phosphoribosyl transferase 1 (HPRT1), and the ribosomal protein L32 (RPL32).

### 3.2. Basal Differences

**Figure 1:**
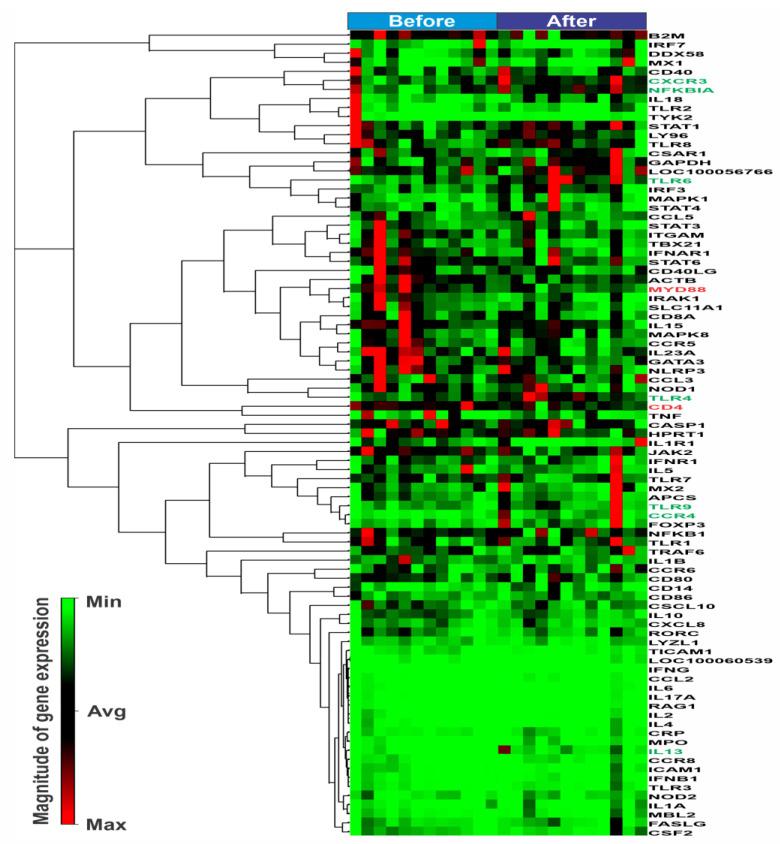
A heatmap of the expression of innate and adaptative immune genes in horses before and after a competition. The differences among the individuals and states (before and after the competition) are shown by differences in color according to the scale for higher (red) or lower (green) gene expression.

The result of the heatmap of the expression of innate and adaptive immune genes in horses before and after the exercise is presented in Figure 1. It is important to note that though the horses were of the same age, breed, and condition, significant variation between individual samples was observed for several genes, as can be seen in the heatmap (Figure 1), even between individuals before exercise, showing differences in the basal gene expression. This could suggest that it is not possible to find statistically significant differences (of biological meaning) in this type of study using a low number of subjects, as is common in RNA-Seq experiments, which usually test 3–4 individuals, due to the high cost of this technique. In addition, when we analyzed the differences in gene expression between fillies and colts before and after the exercise, we observed some sex specific differences (*p* < 0.05) between the differentially expressed genes.

### 3.3. Differentially Expressed Genes

**Figure 2:**
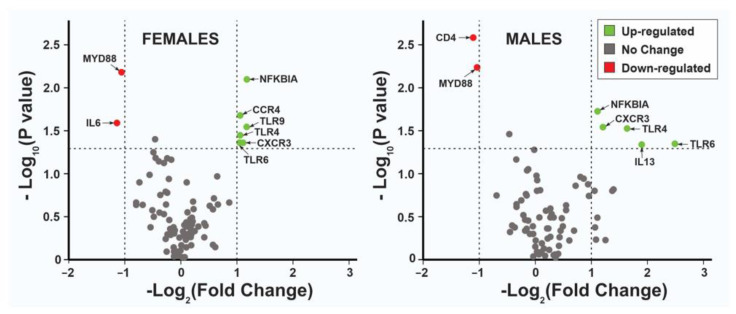
Transcriptional changes of the 84 innate and adaptative immune genes in horses. The horizontal line (dashed) indicates a *p*-value threshold of 0.05. Genes with data points in the upper left (downregulated and red) and upper right (upregulated and green) sections showed greater than the 1-fold regulation and *p*-value thresholds. Genes with identical expression patterns grouped around the 0 fold change and *p*-value.

The most biologically significant genes in this experiment are presented through a volcano plot (Figure 2). This plot compares the statistical significance of the difference relative to the magnitude of the difference for each individual gene using negative base 10 log and base 2 log fold change, respectively.

**Figure 3:**
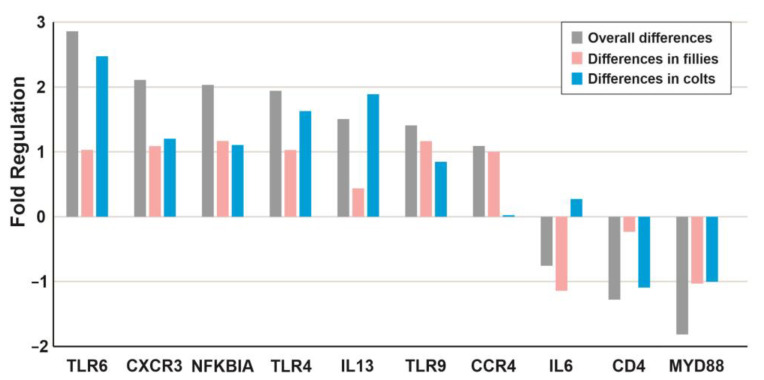
Changes in the transcriptional activity of immune response genes in horses, showing only those that were statistically significant for the overall analysis (both sexes combined) or for each individual sex. The expression before the exercise was set to 0. Significant fold differences were considered to be those greater than +1 or −1.

The results of the transcriptional changes in the innate and adaptive genes showing statistically significant differential expression in horses before and after exercise are presented in Figure 3. The overall analysis showed seven genes to be upregulated and two downregulated (Table 1), with fold changes from 1.09 to 2.88 and −1.28 to −1.81, respectively. Differential expression was observed in seven of the nine genes in females in the overall analysis. The IL13 and CD4 genes did not show statistical differences, but a difference was observed for interleukin 6 (IL6). The IL6 gene did not show significant differences in males or in the overall analysis (Figure 2 and Figure 3). The expression of the males showed differences in seven out of the nine genes detected in the overall analysis, with no differences in TLR9 and CCR4. The level of differences in females ranged from 1.00 to 1.17, while in the males, it ranged from 1.01–2.49 (Figure 3, Table 1). Since the expression level was measured right after the exercise, we expected the changes to be small. Thus, we used the 1.5-fold change as a threshold, but with a P value of 0.05 or lower.

### 3.4. Pathway Analysis

**Figure 4:**
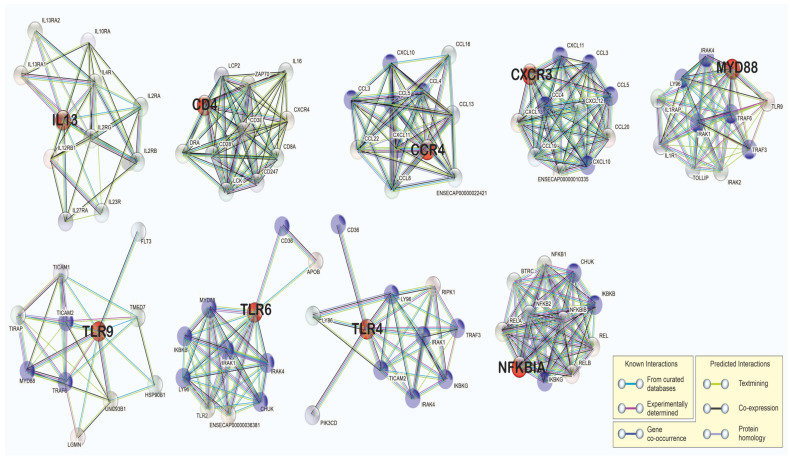
Protein network of the differentially expressed genes (red), showing the main proteins with which, they interact. There were proteins that were present more than once (blue) among these groups of networks. The networks were obtained from the STRING database based on the functional enrichment analysis of the protein–protein interaction networks.

The protein network of the differentially expressed genes is presented in Figure 4. Network analysis of the differentially expressed genes showed that many of the proteins were part of more than one network (Figure 4), except for those in IL13 (upregulated) and CD4 (downregulated), which means that these genes are part of the associated pathways that are activated by acute exercise. MYD88 is part of the protein network of TLR6 and TLR9, as expected, since it is an essential signal transducer in the interleukin-1 and Toll-like receptor signaling pathways.

**Figure 5:**
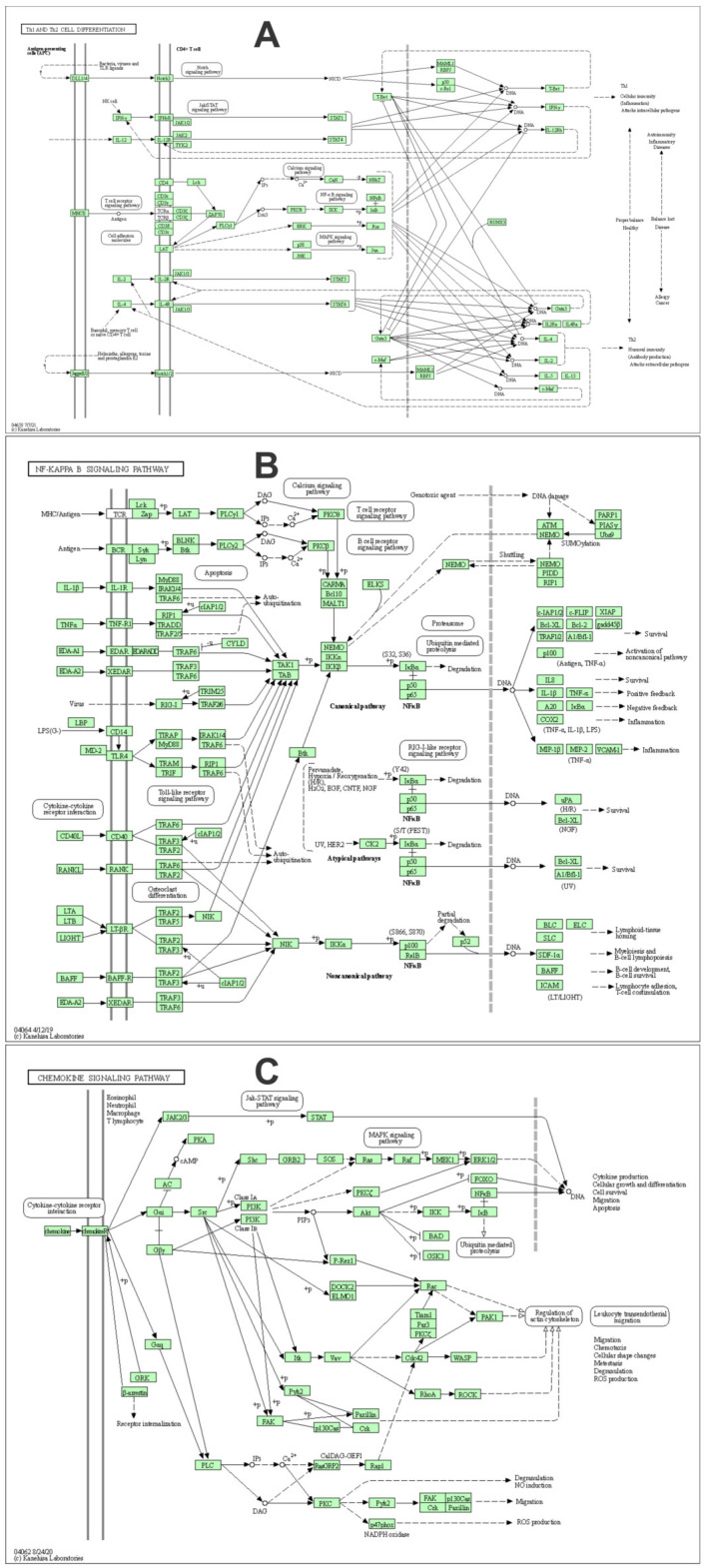
Pathways of the innate and adaptative immune system that contain genes that were differentially expressed by the acute physical exercise in horses. (**A**) The Th1 and Th2 cell differentiation signaling pathways, which are mainly driven by cytokines IL2 and IL4, respectively. (**B**) The NF-kappa B signaling pathway, which is considered as a regulator of innate immunity and links pathogenic signals and cellular danger signals, thus organizing cellular resistance to invading pathogens. (**C**) The chemokine signaling pathway, which is associated with the activation, differentiation, and migration of immune cells [13].

Pathways analysis of the innate and adaptive immune genes that were affected by the acute physical exercise in horses are presented in Figure 5. The three main pathways containing genes that were affected by acute, intense physical exercise were Th1 and Th2 cell differentiation, and the NF-kappa B and chemokine signaling pathways (Figure 5).

## 4. Discussion

Overtraining syndrome (OTS) is the reduction in performance due to excess training and lack of proper recovery, which can lead to chronic deprivation of energy and reduction in the repair of small damage that accumulates over time. In the early stages, OTS can be presented as functional overreaching (FOR), nonfunctional overreaching (NFOR), or full overtraining syndrome, with no clear limits among the three stages [14]. One difference among the three could be based on the duration and the recovery of each episode. For example, if the reduction in performance is for a week or less, and the performance is increased after a resting period, it is likely to be a FOR, while if it lasts for more than a week and the performance after the recovery is the same or worse, it could be a NFOR. If the underperformance persists for several weeks, even after sufficient recovery periods, it is a true OTS [14].

The differentiation of FOR, NFOR, and OTS have been proposed by the detection of changes in the levels of hydroperoxides, plasma antioxidant capacity, red blood cell glutathione, superoxide dismutase, coenzyme Q10, α- and γ-tocopherol, carotenoids lutein, α- and β-carotene, which increased IL1β, IL6, and TNF as well as reduced IL10, basal leptin, and IGF1 response to exercise [14], but a diagnosis cannot be achieved with precision, allowing for possible misdiagnosis. In this sense, the assessment of the expression levels of genes in the pro-inflammatory pathways could be an effective strategy to differentiate among the different stages of OTS and to help optimize the training scheme in horses.

Liburt et al. [15] studied the effect on the gene expression of the incremental exercise test vs. a standing parallel control in the blood and muscle biopsies from four healthy, unfit Standardbred mares. They found that the exercise induced significant upregulation for IFNG, IL1, and TNF in the blood and upregulation for IFNG, IL6, and TNF in the muscle, but no significant changes for IL1 in the muscle or IL6 in blood, after the exercise. All of the cytokine markers of inflammation returned to the preexisting expression levels by 24 h after the exercise test.

Capomaccio et al. [16] carried out a microarray expression analysis to study the effect of strenuous conditions on the immune genes of peripheral blood mononuclear cells at three different timepoints (at rest, just after, and 24 hours after an endurance race). They showed that gene expression differences were only found just after the race. They detected 97 upregulated and 35 downregulated genes in the samples after the race. A validation by RT-qPCR of the top 26 most differentially expressed genes showed fold changes ranging from 1.22 to 4.98 and −1.15 to −2.07, respectively. The genes with the highest fold changes in the innate and adaptative immune system were IL1R2 (4.98), IL18 (2.10), CXCL2 (1.99), IL8 (2.12), SOCS3 (2.596), IL1B (2.10), TLR4 (1.53), CCR2 (2.29), FCER1A (1.73), and IFNAR1 (1.91). Of these genes, we also found TLR4 to be upregulated in our study, but differences from this study could be due to the duration and type of the exercise, which in their case was much longer and slower paced.

Capomaccio et al. [6] carried out the sequencing of transcriptomes of peripheral blood mononuclear cells at two time points: Basal (T1), with the athlete at rest, and immediately at the end of the race (T2), from two Arabian horses. The differential expression analysis showed that the exercise induced gene clusters involved in inflammation, cell signaling, and immune interactions, among other functions, to downregulate CCL4, CCL5, CD4, and CD8 and upregulate many IL6-type cytokines including IL8, IL18, IL1 receptors, and the inducible negative regulator of cytokine signaling, SOCS3. The most highly upregulated gene was IL22A2, and IL22RA2, which regulates shared central pathways, and is a common risk gene for several immune disorders [17]. CD4 was also found to be downregulated in the current study.

Cappelli et al. [18] studied the expression of eight genes by RT-qPCR in 10 athletic horses (five males, five females; mean age ± SD 4.2 ± 2.44 years) before, right after, and 12 h after a race (1000–2400 m) as well as nine untrained mares (aged 10.2 ± 2.4 years). Their results showed that six of the genes (TLR4, IL1b, IL1RII, IL18, IL6, and CEBPB) had higher expression levels in the athletic horses compared to sedentary mares, when both were at rest. However, when comparing the genes before and after the race in athletic horses, they found that only CXCL2, IL8, and CECPB showed differences right after the race but not 12 h after.

Ropka-Molik et al. [19] carried out transcriptome profiling of 12 Arabian horses at three timepoints (conditioning phase, intense gallop phase, and the end of the racing season) as well as in six untrained horses (2.5 years old). These authors found an increase in the number of differentially expressed genes between training periods, with the greatest number of DEGs (440) found between the untrained horses and horses at the end of the racing season. The functional annotation showed that the most upregulated genes in exercise were involved in cell cycle regulation (PI3K-Akt signaling pathway), cell communication (cAMP-dependent pathway), proliferation, differentiation, and apoptosis as well as immunity processes (Jak-STAT signaling pathway). Among the genes with the highest fold change were interleukins (IL18; IL8; IL1R2), chemokines (CXCL2; CCR2; CCL5), and the IFNAR1 gene. The increase in the acute-phase response molecules confirmed that long-term training is a stress factor and that these inflammatory responses play an important role in triggering downstream signaling pathways that are essential for homeostasis maintenance.

Page et al. [20] studied the expression of the genes ALOX5AP, CD14, IL-10, IL-1b, IL-6, IL-8, MMP-1, TLR4, TNF, and TNFSF13B in 77 horses (7–27 years of age) competing in a 160-km endurance ride at the beginning, at two intermediates, and a final timepoint. They found that all genes had an increase in the expression at different times compared to the initial state, which for TLR4, is congruent with our results.

Miglio et al. [10] studied 29 Thoroughbred racehorses of 2 years of age at one month before the start of training, just before training, and 30, 60, and 90 days after the training started, analyzing the hematobiochemical parameters and the expression of eight genes. They found that many of the hematobiochemical parameters changed when comparing T-30 to T-90, and the expression of the IL4, IL6, and OCT1 genes increased, while the one for BCL11A decreased after training.

Cappelli et al. [7] studied common hematological parameters and the gene expression of 11 immune genes in 19, 2-year-old Thoroughbred racehorses (seven males and 12 females) before initiating training, and at 30 and 90 days after. They found that at day 30, IL2, IL4, and IL8 showed increased expression, which was reported to be higher at 90 days compared to 30 days as well as an increase for IL1B and IL3, while IL3 and TGFB1 showed decreased expression at day 90. 

Interleukin 13 (IL13) showed the highest increase in expression after exercise in the current study. This protein is one of the cytokines secreted by Th2, which is a specialized T helper (Th) cell subset differentiated from naïve CD4+ T cells as well as innate lymphoid cells (ILC2s), and granulocytes [21]. IL-13 has been found to be significantly elevated in the serum and affected tissues of patients suffering from many autoimmune diseases including Sjögren’s syndrome, rheumatoid arthritis, and systemic sclerosis, and it plays a critical role in the progression of allergic diseases [22]. However, in a study by Knudsen et al. [21] of women divided into three groups: with obesity, normal-weight with sedentary habits (controls), and endurance athletes, the endurance-trained women had significantly higher IL-13 levels but lower IL-6 levels than obese women, who had higher IL6 levels than the controls. In the same study but a separate cohort of men with normal-weight and sedentary habits (controls), college cross-country runners, and American-football players (samples collected postseason), the male athletes had significantly higher plasma IL13 levels but showed no change in IL6 levels compared to the controls. 

In addition, in the same study by Knudsen et al. [21] it was reported that during endurance training, mice increased the number of oxidative fibers in the muscle and improved mitochondrial respiration, endurance capacity, and glucose tolerance, but IL13-deficient muscle showed defective fatty acid utilization after a single bout of exercise and failed to increase the mitochondrial biogenesis after endurance training, which suggested that all of the metabolic benefits of exercise training required intact IL13 signaling. The increase in the IL13 in horses in the current study due to the exercise could be associated with the activation of IL13 signaling, as seen in the reports by Knudsen et al. [21]. 

CCR4 is another upregulated gene in this study that has been implicated in the pathogenesis of inflammatory diseases because it is expressed in Th2 cells, and their endogenous chemokine ligands are secreted by activated antigen-presenting cells including dendritic cells, monocytes, and macrophages, activating these Th2 cells, which then contribute to the development of inflammation through the production of IL4, IL5, and IL13 [23]. Previous reports have found that CCR4+ Th2 cells are elevated in the airways of asthma patients, and recent studies have associated the upregulation of CCR4 and its binding chemokines CCL17 and CCL22, with enhanced allergic inflammation through a positive feedback loop among the receptor and these ligands [23].

Xiang et al. [24] studied a cohort of five female and 11 male healthy recreational marathon runners (aged 26–57 years) before and one week after a marathon and found that CXCR3 and IRF1, Th1 related genes, were upregulated one week after the marathon. However, CCR2, CCR3, CCR4, CEBPB, GPR44, NFATC2, NFATC2IP, TMED1, IL4, and GATA3, Th2 related genes, were significantly upregulated immediately after the marathon. This suggests a Th1/Th2 imbalance and that transcription factor gene expression associated with Th1 and Th2 cytokine production is affected, which may possibly contribute to an increased susceptibility to post-race infections. 

TLR4, TLR6, and TLR9 are transmembrane proteins, members of the Toll-like receptor (TLR) family whose main function is the recognition of pathogen-associated molecular patterns (PAMPs), which are found on infectious pathogens and activate transcription factors via mediating the synthesis of cytokines required for the establishment of efficient immunity. The innate immune responses are controlled by nuclear factor kappa B subunits and interferon regulatory factors [25]. The specific PAMPs recognized by the TLR family members have been well-characterized: TLR2 homodimers and TLR2–TLR1 and TLR2–TLR6 heterodimers mediate responses to bacterial lipoproteins, peptidoglycan, lipoteichoic acid, and zymosan; TLR3 to double-stranded RNA, a marker of viral infection; TLR4 to bacterial lipopolysaccharide (LPS); TLR5 to bacterial flagellin; TLR7 and 8 to imidazoquinolines and single stranded RNA, respectively; and TLR9 to bacterial DNA [26].

According to numerous studies, TLR4 promotes the inflammatory response in the heart, a mechanism that is critical in the emergence of cardiovascular illnesses, and very recently, it has been shown to influence the apoptosis and endoplasmic reticulum stress-related genes that were activated in the myocardium of C57BL/6 mice subjected to different acute sessions of physical exercise, highlighting the link between TLR4, physical activity, and cardiac responses [27]. For example, Wu et al. [28] demonstrated that chronic aerobic exercise reduced TLR4, and other articles have demonstrated that the beneficial effects of TLR4 downregulation mediated by physical exercise could act in a variety of chronic conditions other than cardiovascular disease such as insulin resistance in people with type 2 diabetes mellitus and obesity, and inflammation [28].

Excessive training, on the other hand, causes trauma in the skeletal muscle, connective tissue, and bone tissue, resulting in the release of pro-inflammatory cytokines that affect the central nervous system, sympathetic nervous system, hypothalamic gonadal hypothalamic axis, and liver [29]. Furthermore, excessive exercise increases the production and release of proinflammatory cytokines induced by TLR4, while also disrupting mitochondrial dynamics, mitophagy, and biogenesis, contributing to a decline in physical performance.

In dendritic cells, TLR7 and TLR9 are activated by RNA and DNA viruses, respectively, which induce the signaling cascade of MYD88 and IRF7 to activate the production of type I IFN, and the MYD88-IRAK4-TRAF6 complex drives NFKB-dependent inflammatory cytokine induction [25]. It is notable that TLR9 signals through different cellular compartments that induce either MYD88-IRF7-dependent type I IFN or MyD88-NF-κB-dependent inflammatory cytokines [30]. TRAF3 has been shown to be incorporated into the MYD88 complex as well as the TRIF complex in TLR4 signaling [25], and the TRAF3–MYD88 complex is then degraded, which causes an inhibition of the MyD88-dependent pathway. This could be the reason why MYD88 was found to be downregulated after exercise in the current study.

The NFKBIA protein interacts with REL dimers to inhibit NF-kappa-B/REL complexes that are involved in inflammatory responses through growth factors and cytokines, which participate in several biological processes, and its deregulation results in pathological processes [31]. In fact, a study discovered that a common NFKBIA variation may change the gene’s expression and is linked to lower estimated glomerular filtration rate (eGFR) at the population level, which may raise the risk of developing type 2 diabetes and impaired renal function [32]. Blocquiaux et al. [33] studied the gene expression and methylation of skeletal muscle to determine the effect of retraining in old (exercise group = 6, 66 ± 5 years and control group = 3, 70 ± 4 years) and young men (five individuals 22 ± 2 years). They found 427 differentially expressed genes between young and older men when they were trained for 12 weeks. Additionally, they found increased expression due to previous training for both young and older muscle, which was partially explained by the retained and novel methylation patterns. This suggests that earlier training periods and resistance training can be effective to rejuvenate the muscles. Interestingly, they found that NFKBIA, which inhibits NF-κB signaling, was downregulated following 12 weeks of training in older men, which could be an indication of maintained inflammation in older muscles. Surprisingly, following detraining and retraining, the NFKBIA expression levels stabilized and were restored to the baseline levels. These findings suggest that age-related low-grade inflammation may actually be improved by repeated strength training, and that previously trained older muscle may be less vulnerable to exercise-induced chronic inflammation. The fact that we found an upregulated expression of NFKBIA in the current study could mean that the training scheme used in young horses is adequate, thus not inducing inflammation. 

CXCR3 promotes the chemotaxis of Th1 cells as well as the activation of eosinophils and mast cells when activated by chemokines CXCL9, CXCL10, and CXCL11 [34]. This is an important inflammation pathway, since Th1 cells produce IFNG, which promotes CXCL10 expression, leading to positive feedback to increase type 1 inflammation. T cell CXCR3 and its ligands CXCL9 and CXCL10 as well as CCR5 and its ligands CCL3 and CCL5 are expressed at higher levels in disorders where Th1 immune responses predominate such as multiple sclerosis and rheumatoid arthritis [35]. The upregulation of the CXCR3 in the current study could suggest that there is an incipient level of inflammation promoted by this and other genes involved in this pathway.

Tossige-Gomes et al. [36], studying 16 young healthy men who underwent high-intensity interval training, found that the frequency of CD4+ and CD19+ cells was unchanged or reduced, and that under superantigenic but not mitogenic stimulation, this high-intensity training increased lymphocyte redox imbalance and decreased lymphocyte proliferation. Our results showed that CD4 was one of the two genes downregulated after acute exercise, which agrees with this report.

The three main pathways containing genes that were affected by acute, intense physical exercise were Th1 and Th2 cell differentiation, and the NF-kappa B and chemokine signaling pathways (Figure 5). Activation of naive CD4^+^ T cells mainly by dendritic cells results in their differentiating into Th1, Th2, Th17, Tfh, and regulatory G cells (Treg), which mediate effective immune responses and leading lineage-determining molecules, interferon-γ (IFNG) and T-bet for Th1, IL-4/IL-5/IL-13; GATA3 for Th2, IL-17/IL-22; RORγt for Th17, IL-21; and Bcl6 for Tfh; and IL-10/ TGFB/IL-35 and Foxp3 for Treg [37]. The Th1 cells are important for cellular immunity, activating, and/or stimulating other immune cells including CD8 T cells, ILC1s, macrophages, and B cells during the process of pathogen elimination, and through the expression of CXCR3, the migration of Th1 cells toward the inflammation sites with pathogen invasion. Th2 is mainly involved in host defense against large extracellular pathogens, producing IL2 and IL4, which activate STAT5 and STAT6, respectively, and the latter expresses GATA3, which also represses the expression of other lineage transcription factors such as T-bet and RORγt [37]. 

The activation of NF-κB is important for regulating immune and inflammatory responses and is divided into the canonical NF-κB pathway. This pathway is involved in almost all aspects of immune responses and responds to diverse stimuli including ligands of various cytokine receptors and pattern-recognition receptors (PRRs). Others include the TNF receptor (TNFR) superfamily members as well as the T-cell receptor (TCR) and B-cell receptor. It is important to note that the noncanonical pathway selectively responds to a specific group of stimuli including ligands of a subset of TNFR superfamily members such as LTβR, BAFFR, CD40, and RANK and appears to have evolved as a supplementary signaling axis that cooperates with the canonical pathway in the regulation of specific functions of the adaptive immune system [38].

In humans, more than 50 chemokines and 19 chemokine receptors have been identified, with the receptors divided into several subfamilies: the CXC, CC, CX3C, and XC chemokine receptors, which play a critical role in inflammatory responses and are associated with immune cell activation, differentiation, and migration [39].

Severe exercise has been shown to lead to tissue trauma that can activate the production of cytokines and stimulate the differentiation of naive Th- cells into Th2 as well as elevating the circulation of stress hormones including cortisol and catecholamines, which can further push the balance toward Th2 lymphocyte response [40,41]. In this sense, Lancaster [42], Wang and Chen [43], and Dong et al. [44] demonstrated that excessive exercise leads to increased secretion of inflammatory cytokines and chemokines in the peripheral blood, enhancing the Th2 responses that could cause a Th1–Th2 imbalance in immunological regulation and a decrease in cellular immunity. In a chronic stage, this could lead to an increase in infection, especially of respiratory pathogens. However, there are not enough studies that help to elucidate the genetic and physiological mechanisms that lead to or regulate/prevent OTS, and the current study is a contribution to find the initial steps that can progress toward OTS.

## 5. Conclusions

In the current study we found that the three main pathways containing genes that were affected by acute, intense physical exercise were Th1 and Th2 cell differentiation, and the NF-kappa B and chemokine signaling pathways, suggesting the activation of proinflammatory responses as the result of the stress from the acute exercise. Even though the changes in the differentially expressed genes were subtle, and a period of rest can reset these changes, we believe that excess of training, along with the mechanisms of cell damage produced by OTS, can lead to detrimental effects on the immune response. We also propose that gene expression could be used to assess any indication of OTS.

## Figures and Tables

**Table 1:** Differentially expressed mRNAs of the innate and adaptative immune genes in horses before and after a high intensity training session. Basal expression (before competition) was set to 0 and the expression fold difference was calculated from that level up or down. The overall analysis changes were calculated using all of the individuals regardless of sex. Changes were calculated for each sex separately.

Symbol	Name	RefSeq	Overall Fold Change	Overall *p*-Value	Female Fold Change	Female *p*-Value	Male Fold Change	Male *p*-Value
TLR6	Toll-like receptor 6	NM_001257142	2.88	0.003	1.04	0.044	2.49	0.045
CXCR3	C-X-C chemokine receptor type 3-like	XM_001493611	2.12	0.001	1.10	0.044	1.21	0.029
NFKBIA	Nuclear factor of kappa light polypeptide gene enhancer in B-cells inhibitor, alpha	XM_005603476	2.04	0.000	1.17	0.008	1.11	0.019
TLR4	Toll-like receptor 4	NM_001099769	1.95	0.001	1.03	0.036	1.64	0.030
IL13	Interleukin 13	NM_001143791	1.51	0.032	0.44	0.407	1.90	0.048
TLR9	Toll-like receptor 9	NM_001081790	1.41	0.038	1.17	0.029	0.85	0.440
CCR4	Chemokine (C-C motif) receptor 4	XM_005600863	1.09	0.034	1.00	0.021	0.02	0.612
IL6	Interleukin 6 (interferon, beta 2)	NM_001082496	−0.76	0.159	−1.14	0.026	0.26	0.547
CD4	T-cell surface glycoprotein CD4-like	XM_001497051	−1.28	0.034	−0.23	0.517	−1.10	0.003
MYD88	Myeloid differentiation primary response protein MyD88-like	XM_001488549	−1.81	0.000	−1.03	0.007	−1.01	0.006

## Data Availability

The dataset generated in this study can be requested from the corresponding authors.
